# In Vivo Bio-distribution and Efficient Tumor Targeting of Gelatin/Silica Nanoparticles for Gene Delivery

**DOI:** 10.1186/s11671-016-1409-6

**Published:** 2016-04-12

**Authors:** Xueqin Zhao, Jun Wang, SiJie Tao, Ting Ye, Xiangdong Kong, Lei Ren

**Affiliations:** College of Life Sciences, Zhejiang Sci-Tech University, Hangzhou, 310018 People’s Republic of China; Department of Biomaterials, College of Materials, Xiamen University, Xiamen, 361005 People’s Republic of China; Fujian Collaborative Innovation Center for Exploitation and Utilization of Marine Biological Resources, Xiamen University, Xiamen, 361005 People’s Republic of China

**Keywords:** Cellular uptake and transfection, In vivo and ex vivo imaging, In vivo transfection, Non-viral vector, Surface modification

## Abstract

The non-viral gene delivery system is an attractive alternative to cancer therapy. The clinical success of non-viral gene delivery is hampered by transfection efficiency and tumor targeting, which can be individually overcome by addition of functional modules such as cell penetration or targeting. Here, we first engineered the multifunctional gelatin/silica (GS) nanovectors with separately controllable modules, including tumor-targeting aptamer AGRO100, membrane-destabilizing peptide HA2, and polyethylene glycol (PEG), and then studied their bio-distribution and in vivo transfection efficiencies by contrast resonance imaging (CRI). The results suggest that the sizes and zeta potentials of multifunctional gelatin/silica nanovectors were 203–217 nm and 2–8 mV, respectively. Functional GS-PEG nanoparticles mainly accumulated in the liver and tumor, with the lowest uptake by the heart and brain. Moreover, the synergistic effects of tumor-targeting aptamer AGRO100 and fusogenic peptide HA2 promoted the efficient cellular internalization in the tumor site. More importantly, the combined use of AGRO100 and PEG enhanced tumor gene expression specificity and effectively reduced toxicity in reticuloendothelial system (RES) organs after intravenous injection. Additionally, low accumulation of GS-PEG was observed in the heart tissues with high gene expression levels, which could provide opportunities for non-invasive gene therapy.

## Background

Gene delivery to cells can be accomplished by using viral and non-viral vectors. Regarding safety concerns, the use of viral vectors has been limited, leading to the evaluation and development of alternative vectors based on non-viral systems. However, to successfully translate the use of non-viral vectors from laboratories to clinics, numerous barriers such as transfection efficiency, tumor targeting, and blood clearance must be overcome to achieve efficient gene delivery [1]. The barriers can be individually overcome by the addition of functional modules such as conjugation of moieties for cell penetration or targeting. Some ligands such as cell-penetrating peptides Tat, fusogenic peptide HA2, and folic acid designed or attached to the surface of gene vectors displayed good intracellular trafficking into target cells [2–4]. It has been reported that HA2 could influence macrophage uptake and the distribution of antisense oligonucleotides (ONs) [5, 6]. Nucleic aptamers are potentially well-suited for the therapeutic targeting of drug encapsulated controlled release polymer particles in a cell- or tissue-specific manner [7, 8]. G-quadruplex aptamer, AGRO100, can strongly bind to nucleolin, a multifunctional protein expressed at high levels in cancer cells [9]. It is currently being evaluated in phase I clinical trials as the first nucleic acid-based aptamer for use in cancer treatment in humans. Besides, modification by a hydrophilic polymer such as polyethylene glycol (PEG), polyethylene oxide, glucan, and dendrimer is generally a useful strategy for reducing the reticuloendothelial system (RES) uptake, thus increasing the circulation time of administered NP-linked drugs because nanostructured drugs administered systematically can be taken up primarily by the RES through the phagocytic pathway [10–13]. PEGylation nanoparticles exhibit long circulating properties and are preferentially distributed in tumors [14]. The combination of multiple functional modules into a single nanocarrier can increase the intracellular delivery of many drugs, genes, and proteins, which might enhance their therapeutic efficacy [15–17].

Protein-based carriers commonly show low levels of RES clearance, leading to improved pharmacokinetic properties [18]. Gelatin, as a natural protein, has excellent biocompatibility and gelification properties. The strong dependence of gelatin ionization with pH makes it a competitive candidate for DNA encapsulation [19]. We previously synthesized a series of siloxane crosslinked gelatin/silica nanovectors (GS NPs) with controlled size and surface charge through a two-step sol–gel process and investigated their cellular internalization and transfection efficacy in vitro [20–23]. It was found that GS NPs are low cytotoxic biomaterials with strong DNA encapsulation ability as well as considerable transfection efficiency, which was nearly 70 % compared to the commercial transfection reagent Lipofectamine TM [20]. Furthermore, the grafting of fusion peptides (Tat, R8, or HA2) onto GS NPs resulted in a synergistic effect on cellular internalization and transfection efficacy [22, 23]. These results indicated that these GS NPs have excellent properties as highly potent and non-toxic intracellular delivery systems, rendering them promising DNA vehicles to be used as non-viral gene delivery systems. However, the in vitro and in vivo transfection efficiencies do not always correlate, making the translation of positive results in cell culture to positive results in animal studies even more difficult.

Herein, we extended the previous work to the synthesis of a multifunctional gelatin/silica gene delivery system with separately controllable functions, including a polymer matrix for gene stabilization/controlled release, protein HA2 for cell penetration, aptamer AGRO100 for tumor targeting, and surface-bound PEG for enhancing circulatory time. In vitro and in vivo studies were performed to evaluate the effectiveness of this system. Systematic studies were performed to evaluate the effect of the surface properties on mediating gene transfection, which may help in designing functionalized non-viral vectors in the future.

## Methods

### Materials

Gelatin (bloom number: 240–270, pH 4.5–5.5) was purchased from Bio Basic Inc. (Amherst, New York, USA). 3-glycidoxypropyl-trimethoxysilane (GPSM) and 3-aminopropyl-trimethoxysilane (APTMS) were purchased from Acros Organics (Geel, Belgium). N-succinimidyl 3-(2-pyridyldithio) propionate (SPDP) was purchased from Pierce Biotechnology (Waltham, MA, USA). A PEG polymer of molecular weight 2000 Da with a terminal amine and carboxylic group (NH_2_-PEG-COOH) was purchased from Beijing Kaizheng Biotech Development Co. Ltd. (Beijing, China). HA2 peptide (GLFGAIAGFIENGWEGMIDGC) was provided by Shanghai GL Biochem Ltd. (China). Luciferase plasmid pGL3 and luciferase assay system were purchased from Promega (Madison, WI, USA). BCA protein assay kit was obtained from Pierce Biotechnology. Plasmid DNA was purified using the Qiagen plasmid maxi kits (Hilden, Germany) according to the manufacturer’s instructions. The dyes of rhodamine B isothiocyanate (RITC), propidium iodide (PI), and tetramethylrhodamine isothiocyanate (TRITC) as well as DNA aptamer (AGRO100, ggT ggT ggT ggT TgT ggT ggT ggT ggT TTT TTT TTT TT) were purchased from Sangon Biotech Co., Ltd. (Shanghai). All materials used were of analytical grade and used without further purification.

### Material Synthesis and Characterization

Amino-functionalized gelatin/silica nanovectors (GS NPs) were first prepared according to previously reported methods [20]. Total amino group (-NH_2_) levels on the surface of GS NPs were quantitatively determined by using the ninhydrin colorimetric reaction. The amount was about 0.642 mmol/g. We conjugated RITC to GS NPs surface based on the reaction between the isothiocyanate group of RITC and the primary amino group of GS NPs. RITC (0.3 mg) was added to 50 mg of GS NPs in pH 8.0 phosphate buffered saline (PBS) and incubated on a rotator for 2 h at room temperature. After purification by centrifugation, NH_2_-PEG-COOH was linked to the surface of GS NPs by the use of the coupling reagents, ethyl-3-(dimethylaminopropyl) carbodiimide (EDC) and N-hydroxy sulfo-succinimide (NHS). Twenty milligrams of NHS-activated PEG was dissolved in 20-mM dimethyl sulfoxide and added to 50 mg of GS NPs in pH 7.5 PBS. The mixture was incubated for 4 h at room temperature with mixing to fabricate GS-PEG NPs. After purification by centrifugation, we further conjugated the PEG with a heterobifunctional crosslinker (3.3 mg), N-succinimidyl 3-(2-pyridyldithio) propionate (SPDP), via the N-hydroxysuccinimide ester in pH 8.0 PBS for 2 h with mixing. Five OD of sulfhydryl-containing DNA aptamer (AGRO100) were attached to this linker via a disulfide bond in pH 8.0 PBS to obtain the bi-functional GS-PEG-Apt NPs. After purification by centrifugation, 12 mg of HA2 peptide was linked to the GS NPs surface by using the coupling reagents, EDC and NHS, to fabricate the tri-functional GS-HA2/PEG-Apt NPs.

For transmission electron microscopy (TEM), samples were prepared by drying one drop of nanoparticle suspension in distilled water on carbon-coated copper grids. Particle size and surface charge were measured by a Nano-ZS Zetasizer dynamic light scattering detector (Malvern Instruments, UK). DNA loading of GS NPs was first accomplished by incubation with the luciferase plasmid DNA (pGL3) for 1 h at room temperature. The procedure was as described for synthesis. DNA condensation ability was examined by gel retardation assay, and DNA retardation was observed by irradiation with a gel documentation system (Tanon GIS-2008, China).

### Cytotoxicity

The cytotoxicity of GS, GS-PEG-Apt, and GS-PEG/HA2-Apt NPs was evaluated against A549 cells using MTT assay. Briefly, A549 cells (1 × 10^4^ cells/well) were seeded in polystyrene 96-well culture plates and incubated for 24 h till the 70 % confluence. The culture medium was then replaced by 100 μl of serum-free DMEM medium containing nanoparticles (100–600 mg/mL). Cells treated with medium only served as a negative control group. After 24 h co-incubation, cells were washed with PBS and incubated with 20 μL of MTT solution (5 mg ml^−1^ in PBS buffer) for 4 h at 37 °C. Then, MTT solution was removed, and 100 μL of DMSO was added to dissolve the blue formazan crystal produced by proliferating cells. The plate was incubated for additional 30 min before determination at 570 nm using a spectrophotometric plate reader (Bio-tek ELX800, USA). All experiments were performed in quadruplicate, and the relative cell viability (%) was expressed as a percentage relative to the untreated control cells.

### In Vitro Cellular Uptake Studies

Cellular uptake was quantitatively examined using flow cytometry. Human lung denocarcinoma A549 cells and fibroblast 3T3 cells (1.5 × 10^5^ cells/well) were seeded in 12-well plates and cultured overnight in containing 10 % FBS Roswell Park memorial institute (RPMI) 1640 medium and Dulbecco’s modified Eagle’s medium (DMEM), respectively. The cells were then treated with GS NPs/DNA, GS-PEG-Apt NPs/DNA, GS-PEG/HA2-Apt NPs/DNA-PI, liposome/DNA-PI, or naked DNA-PI in serum-free RPMI 1640 and serum-free DMEM at 37 °C for 8 h. A single cell suspension was then prepared by trypsinization, washed with PBS, and resuspended in ice-cold PBS. The fluorescent cells with PI incorporated in NPs were then counted from 10,000 cells by using an EPICS XL flow cytometer (Beckman Coulter, Brea, CA, USA) in the FL3 channel. Data analysis was performed with EPICS XL flow cytometer software, and analytical gates were chosen as 1 % of control cells falling within the positive region.

### In Vitro and In Vivo Transgene Expression

Prior to the transfection experiments, the optimal Nanovector/pDNA weight ratio (N/P ratio) to obtain complexes that did not form large aggregates was determined by mixing a fixed amount of plasmid with various amounts of NPs. In general, the transfection complexes were formed by pipetting an equal volume of plasmids into a NP suspension and mixing rapidly. The final DNA concentration of the complexes was 200 μg mL^−1^ for in vivo studies and 10 μg mL^−1^ for in vitro studies.

For in vitro transfection, A549 cells and 3T3 cells (1.5 × 10^5^ cells/well) were incubated in triplicate for 16 h at 37 °C in 5 % CO_2_ before the addition of the DNA complexes. The culture medium was then replaced by 1-mL serum-free RPMI 1640 and serum-free DMEM which contain DNA complexes for 8 h of incubation. After removing the plasmid complexes, the cells were post-incubated for 36 h in medium containing serum. The luciferase expression in cells was quantified with 20 μL of centrifuged lysate supernatant using Luciferase Assay System according to the manufacturer’s instruction. Light emission was measured and relative light units (RLU) mg^−1^ protein determined by using a FB12 luminometer (Berthold, Germany). Protein concentration was determined using the PIERCE BCA protein assay kit according to the manufacturer’s protocol.

For in vivo transfection, the GS-PEG/pGL3, GS-PEG-Apt/pGL3, and GS-PEG/HA2-Apt/pGL3 NPs were injected into the tail vein of BALB/c mice at a dose of 50 μg DNA/mouse. Forty-eight hours following i.v. injection, mice were euthanized and major organs (including the brain, heart, liver, spleen, lung, and kidney) were removed and carefully washed with distilled water and homogenized in 1 mL of lysis reagent using a DY89-II tissue homogenizer. The homogenate was centrifuged at 14,000*g* for 20 min at 4 °C. Luciferase activity and cellular proteins in the supernatant were quantified by a Luciferase Assay System and the PIERCE BCA protein assay kit. The results are expressed as light unit mg^−1^ protein.

### Bio-distribution Studies

Bio-distribution was studied in 6- to 8-week-old nude mice. The mice were maintained in a specific pathogen free house. All animal experiments were performed in compliance with the institutional ethics committee regulations and guidelines on animal welfare. Tumor growth was monitored daily until it reached the acceptable sizes. The mice were divided into three groups for passive and active targeting studies. Two milligrams of RITC-labeled nanoparticles (GS-PEG, GS-PEG-Apt, and GS-PEG/HA2-Apt) were dispersed in 100 μL physiological saline solution and injected into the tail vein. The fluorescence intensities of the nanoparticle solutions were determined before administration to calibrate the TRITC concentration in the solution. One mouse was injected with an equal volume of physiological saline solution as a control. The mice were then anesthetized. Images were acquired by CRi Maestro™ in vivo imaging system. The excitation wavelength was 555 nm, and emission spectra were obtained from 500 to 800 nm.

For ex vivo imaging, after in vivo imaging acquisition, mice were euthanized and major organs were rapidly removed. In addition to direct organ imaging on a non-fluorescent board, organs were grouped for semiquantitative comparison. Photons emitted from tissues were quantified as the sum of all detected photon counts from organs and presented as photons/s using CRi Maestro™ in vivo imaging system.

## Results and Discussion

### Synthesis and Characterization of Nanoparticles

**Fig. 1:**
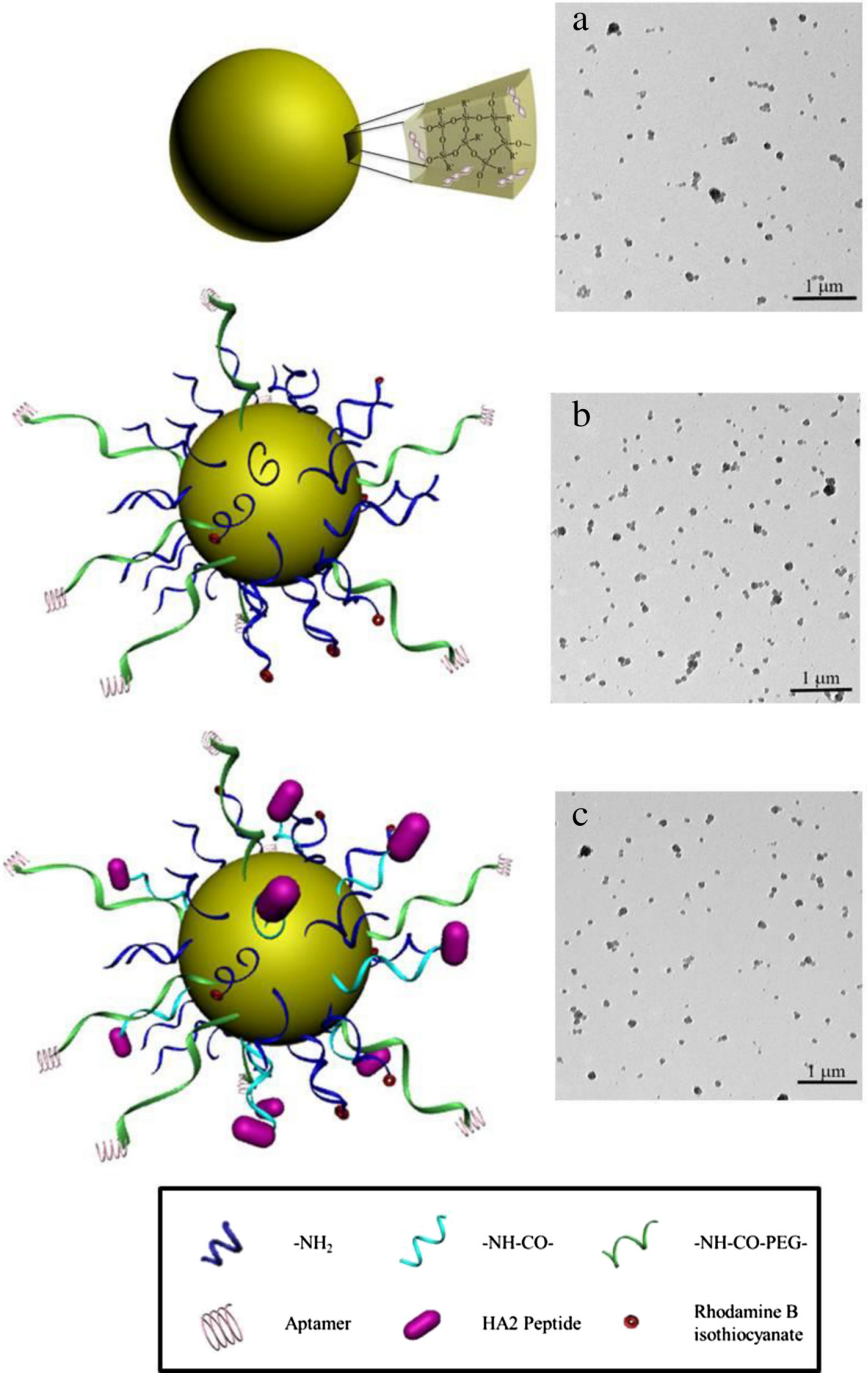
Schematics and TEM images of GS nanoparticles coated with PEG (**a**), nanoparticles coated with PEG and aptamer (**b**), and nanoparticles coated with PEG, HA2 peptide, and aptamer (**c**)

**Fig. 2:**
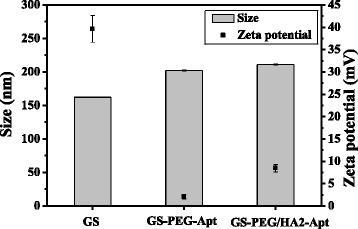
Size and surface charge of GS, GS-PEG-Apt, and GS-PEG/HA2-Apt NPs

Various formulation factors and characteristics of nanoparticles could influence their intracellular delivery [24]. Particles with a size of less than 400 nm present with enhanced permeability and retention effects, which are necessary characteristics for accumulation in solid tumor [25]. Hence, the hydrodynamic diameter and surface charge of the particles were determined by dynamic light scattering measurement. The nanocarriers displayed a well-dispersed spherical structure (Fig. 1). As depicted in Fig. 2, the size of bare GS was 162.3 ± 0.6 nm, while GS-PEG-Apt and GS-PEG/HA2-Apt NPs exhibited relatively larger hydrodynamic diameters of 205.6 ± 2.4 nm and 214.9 ± 2.1 nm, respectively, due to the surface functional layer over the particle. Furthermore, after PEG grafting, the zeta potential of GS-PEG-Apt and GS-PEG/HA2-Apt NPs switched to 2.1 and 8.5 mV, respectively, while that of bare GS NPs was +39.6 mV.

**Fig. 3:**
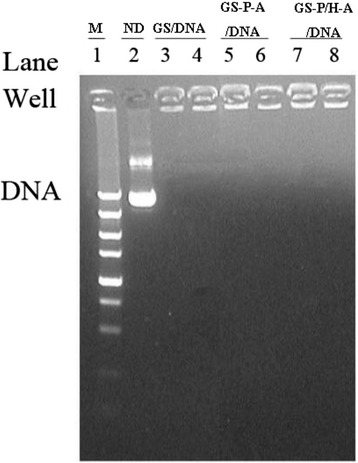
Gel electrophoresis assay of NP/DNA complexes. Lane *1*: marker; Lane 2: naked DNA (ND); Lanes 3–4: GS/DNA complexes; Lanes 5–6: GS-PEG-Apt/DNA complexes; Lanes 7–8: GS-PEG/HA2-Apt/DNA complexes. Complexes with GS/DNA weight ratio of 200 (lanes *3*, *5*, and *7*) and 100 (lanes 4, 6, and 8) were investigated in deionized water

The condensation ability of nanoparticles with plasmid DNA (pDNA) was evaluated by using agarose gel electrophoresis assay. As shown in Fig. 3, free pDNA (lane 2) moved to its usual position, while pDNA was readily entrapped in the sample holes at an N/P weight ratio of 50–200 (lanes 3 to 8). This complete retardation of the pDNA demonstrates that GS-PEG-Apt and GS-PEG/HA2-Apt NPs have strong ability to condense DNA molecules efficiently and thus can be used for in vitro gene delivery.

**Fig. 4:**
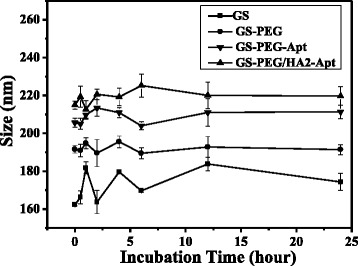
Hydrodynamic size changes of the GS coated with PEG, aptamer, and HA2 peptide incubated in PBS plus 10 % FBS at 37 °C for 24 h

The net surface charge of the nanoparticles also plays a critical role in the clearance of the nanoparticles from the animal body due to different adsorption effects on physiological lipoproteins in the systemic circulation [26]. Hence, we simply mimicked the body environment by adding FBS in the culture medium to evaluate the strength of the nanovectors in the biological environment. As showed in Fig. 4, naked GS size increased by 12.5 %, while PEG-modified nanoparticles increased by less than 5 %, exhibiting long serum stability. The presence of PEG may increase the nanoparticle’s colloidal stability through steric hindrance and provide non-fouling properties [27].

### Cell Compatibility

**Fig. 5:**
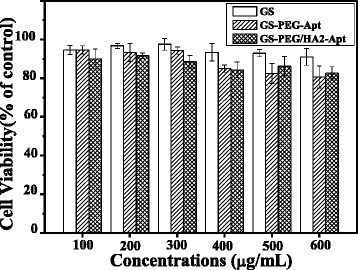
Normalized dose-response for cell viability of GS, GS-PEG-Apt, and GS-PEG/HA2-Apt NPs by MTT assay. Bars represent the corresponding standard deviations (*n* = 6)

The cytotoxicity associated with NPs has always been a concern in their use as gene vectors for gene therapy. As shown in Fig. 5, the cell viability is above 80 % for GS, GS-PEG-Apt, or GS-PEG/HA2-Apt NP groups within the tested concentration range, and the cytotoxicity appeared dose-dependent. Compared with GS NPs, the slight decrease of cell viability might be due to the increased inhibition of NF-κB signaling of conjugated aptamer AGRO100 [9]. Besides, GS-aptamer NPs have a higher level than our previous peptide-GS NPs [20, 23]. Within the tested concentrations up to 600 mg/mL, the NPs can be considered comparatively non-cytotoxic.

### In Vitro Cellular Uptake and Transgene Expression

In vitro optimization is indispensable to set up effective delivery systems in vivo. Hence, we first determined in vitro cellular uptake and transgene expression using the human lung cancer cell line, A549, as a cancer model. The in vitro intracellular uptake was quantitatively analyzed by FCM using the red emitting PI incorporated in NPs as a marker. In vitro transfection efficiency was determined by assessing the luciferase activities in A549 cells and common 3T3 cells, using Lipofectamine (LF) as the positive control. In order to analyze the tumor targeting efficiency of nanovectors quantitatively, *targeting* R was defined as the ratio between uptake or transfection amount of A549 cancerous cells and normal cell 3T3.

**Fig. 6:**
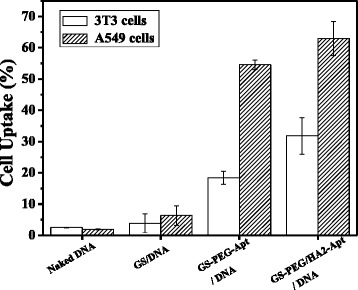
Cellular uptake of PI by A549 and 3T3 cells incubated with DNA-PE, GS/DNA-PE, GS-PEG-Apt/DNA-PI, and GS-PEG/HA2-Apt/DNA-PI NPs for 4 h, as determined by flow cytometry

The different cell uptake percentages resulting from the differently grafted group and cell types are displayed in Fig. 6. All nanoparticles showed an optimized cell uptake by the A549 cells compared with 3T3 cells, especially GS-PEG-Apt and GS-PEG/HA2-Apt presented a five- to tenfold increased uptake due to a receptor-mediated targeting to A549 cell. The increased uptake amount indicates that the peptide-GS nanocarrier internalization is not influenced by surface adsorption. Moreover, the modification with aptamer and HA2 (GS-PEG/HA2-Apt) could enhance the cellular uptake by 15–73 % in comparison with GS-PEG-Apt, which is due to the fact that induced HA2 peptide mediates cell membrane destabilization, further enhancing endosomal escape and increasing cellular distribution [28]. Moreover, the uptake targeting ratio of the bi-functional GS-PEG-Apt was the highest (2.97), followed by GS-PEG/HA2-Apt (1.98) and GS-PEG (1.65).

**Fig. 7:**
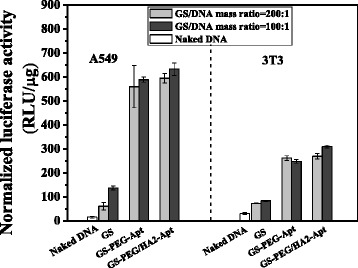
Efficiency of nanoparticle (GS, GS-PEG-Apt, and GS-PEG/HA2-Apt)-mediated in vitro transfection of luciferase plasmid pGL3

The transfection efficiency of three nanovectors (GS-NPs, GS-PEG-Apt, and GS-PEG/HA2-Apt) in two N/P ratios (100 and 200) is presented in Fig. 7. The expression levels of pGL3 delivered by cationic GS and DNA electrostatic complexes were low and similar in both cell types due to their accumulation at the nuclear membrane after cellular internalization. GS-PEG-Apt and GS-PEG/HA2-Apt enhanced gene expression by three- to ninefold over GS NPs and naked DNA, inferring that aptamer AGRO100 increased the transfection efficiency. Especially, after optimization via AGRO100 and HA2, GS-PEG/HA2-Apt in each cell line exhibited a synergistic effect on transfection efficiency. Besides, the expression levels of pGL3 delivered by GS-PEG-Apt and GS-PEG/HA2-Apt NPs in A549 cells were two to three times higher compared to that in the common 3T3 cells, demonstrating that the aptamer AGRO10 modification enhanced the transfection efficiency by generating a targeting effect. In addition, the tri-functional GS-PEG/HA2-Apt presented a higher relative transfection ratio R (2.21) than GS-PEG-Apt (2.13) when the N/P ratio was 200:1, but this was reversed at a N/P ratio of 100:1, which may be explained by a lower cellular uptake targeting of GS-PEG/HA2-Apt NPs.

### In Vivo Bio-distribution and Gene Expression

The therapeutic efficiency of a vector does rely not only on its intrinsic activity but also on transfection efficiency at the targeted site. In this study, a heterobifunctional PEG was employed for conjugation of aptamer AGRO100, taking advantage of PEG’s potential to minimize binding to plasma proteins, thus reaching tumor cells. Additionally, HA2 was introduced to improve the transfection efficiency of nanoparticles in vivo. Thus far, there is no report of in vivo tumor targeting of multifunctional GS nanovectors via intravenous injection.

#### Bio-distribution and Tumor Targeting

**Fig. 8:**
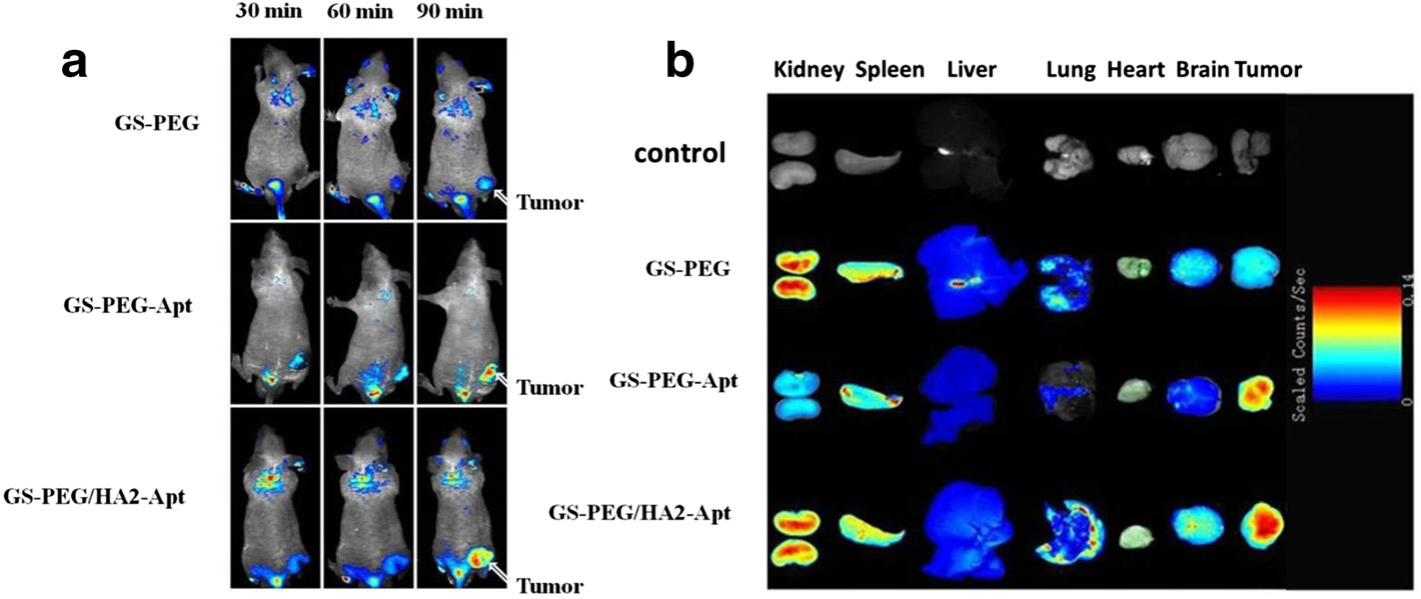
**a** In vivo fluorescence images of A549 tumor-bearing BALB/c mice at different time points post tail-vein injection of rhodamine-labeled GS-PEG, GS-PEG-Apt, and GS-PEG/HA2-Apt NPs. **b** Ex vivo fluorescence images of excised organs at 1.5 h post tail-vein injection

One major pitfall of in vitro studies is that they cannot be reproduced in vivo. GS-PEG, GS-PEG-Apt, and GS-PEG/HA2-Apt NPs were labeled with RITC, and 2 mg/mouse was intravenously administrated into A549 tumor-bearing mice. As shown in Fig. 8, our NPs accumulated dominantly in highly perfused organs such as the liver and spleen. Moreover, the uptake of GS-PEG/HA2-Apt NPs in tumor sites was most obvious, followed by GS-PEG-Apt and GS-PEG during 90 min. This phenomenon might be due to the fact that the surface coverage of NPs altered the characteristics of their distribution in vivo [29]. Firstly, PEGylation could endow NPs a hydrophilic protective layer to repel the protein absorption, further reducing NPs elimination by the RES. Thus, the NPs possessed a good passive targeting effect. Correspondingly, three PEG-functional NPs were present in tumors at 60 and 90 min after injection. Secondly, aptamer AGRO 100 on the surface of the NPs could be recognized and bound with nucleolin-positive A549 tumor cells, later resulting in an increased level of intracellular delivery of NPs to the tumor site. Hence, modifying AGRO 100 on the NPs endows them with tumor-targeting specificity. Thus, more GS-PEG-Apt and GS-PEG/HA2-Apt were found in tumors (Fig. 8). Thirdly, HA2 peptide DNA could help NPs escape quickly from the endosome, improving the efficiency of tumor uptake.

**Fig. 9:**
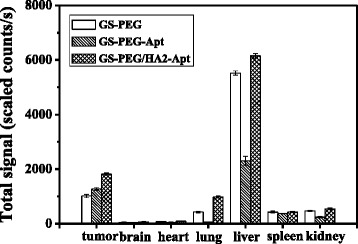
Semiquantitative bio-distribution of GS-PEG, GS-PEG-Apt, and GS-PEG/HA2-Apt NPs in nude mice determined by the averaged fluorescence intensity of the main organs

To further investigate the distribution of the three types of surface-modified NPs in various organs, the animals were euthanized immediately after intravenous injection of GS-PEG, GS-PEG-Apt, and GS-PEG/HA2-Apt at the 90-min time point, and signal intensity from the dissected tissues was quantified as the sum of all detected photon counts per second within the region of interest. As shown in Fig. 9, more than 60 % of nanoparticles mainly accumulated in RES organs (the liver, lung, and spleen) due to a higher circulating blood passing and small quantities (1.5–2.1 %) were detected in the brain and heart. The overall amounts of NPs in tissues were in the order of liver > tumor > kidney, spleen, lung ≥ heart, brain. These observations corroborated the results that the PEG coverage leads to an improved systemic distribution and to the lighting of the most vascularized areas such as the lower limb and liver, which permanently shelters about 10 % of the total blood [29, 30]. Moreover, the accumulation of GS-PEG-Apt and GS-PEG/HA2-Apt in tumor significantly increased by 25.7 and 78.8 %, respectively, when compared with bare GS-PEG (Fig. 9). The high tumor accumulation resulted from the long blood circulation time, the specific high tumor-binding affinity of AGRO 100-functionalized NPs, and the synergistic effect of HA2 and AGRO 100. Additionally, it is interesting to note that, at 90 min, the maximum accumulation of GS-PEG-Apt in the liver was only about half of the other two NPs. This may be attributed to the combined effect of good tumor targeting of AGRO 100 and the relatively higher number of PEG molecules with flexibility, leading to the lower stealthiness to the RES organs.

#### Quantitative In Vivo Gene Expression

**Fig. 10:**
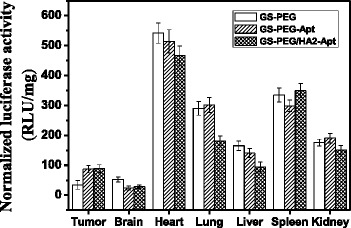
Quantitative evaluation of gene expression in vivo. Luciferase expression 48 h after administration of GS-PEG/DNA, GS-PEG-Apt/DNA, and GS-PEG/HA2-Apt NPs into BALB/c mice at a dose of 50 μg/mouse

Using GS-PEG/pGL3, GS-PEG-Apt/pGL3, and GS-PEG/HA2-Apt/pGL3 complexes for in vivo transfection experiments, the transfection efficiencies in the principal organs were measured at 48 h after intravenous injection (Fig. 10). The amount of pGL3 used was 50 μg/mouse. The luciferase expression of the GS-PEG-Apt/pGL3 and GS-PEG/HA2-Apt/pGL3 NPs in the excised tumors was 86 and 89 U mg^−1^ protein, respectively, about threefold higher than that of the GS-PEG/pGL3 NPs (34 U mg^−1^ protein). Furthermore, relatively low luciferase expression of GS-PEG-Apt/pGL3 and GS-PEG/HA2-Apt/pGL3 NPs was recorded in the brain, heart, and liver when compared with that of GS-PEG/pGL3, whereas the spleen and kidney levels did not markedly changed. Interestingly, in vivo transfection efficiencies of the three nanovectors were inconsistent in their bio-distribution due to tissue-specific differences. This finding demonstrates that the modification of tumor-targeting moieties might be necessary to increase tumor gene expression specificity, and incorporating PEG could effectively reduce toxicity in RES organs after intravenous injection. For all three types of NPs, gene expression in the heart was the highest (33 % in all), which raises a concern regarding cardiovascular diseases, and provides some opportunities for non-invasive gene therapy for specific diseases such as heart failure.

## Conclusions

The clinical application of designed non-virus gene carriers is impeded by inconsistent efficacy in vitro and in vivo. In this study, a gelatin/silica gene delivery system optimized via functional modules (tumor-targeting aptamer AGRO 100, HA2, and PEG) was developed and evaluated for effectiveness in vitro and in vivo. The results demonstrated that the synergistic effects of AGRO 100 and fusogenic peptide HA2 as well as PEG could efficiently promote cellular internalization, in vitro transfection activity, and tumor targeting. However, the modification of aptamer AGRO 100 and PEG might be necessary to increase tumor gene expression specificity and to effectively reduce toxicity in RES organs after intravenous injection. Moreover, we observed a low accumulation of GS-PEG in the heart tissues with high gene expression levels, which could provide some opportunities for non-invasive gene therapy.
